# Correction: Preparation and photocatalytic application of a S, Nd double doped nano-TiO_2_ photocatalyst

**DOI:** 10.1039/c8ra90087f

**Published:** 2018-11-14

**Authors:** Shuo Wang, Xinling Ao, Liming Bai

**Affiliations:** College of Chemistry and Chemical Engineering, Qiqihar University Qiqihar Heilongjiang 161006 P. R. China 872349111@qq.com +86 15561926110

## Abstract

Correction for ‘Preparation and photocatalytic application of a S, Nd double doped nano-TiO_2_ photocatalyst’ by Shuo Wang *et al.*, *RSC Adv.*, 2018, **8**, 36745–36753.

In the published article, Liming Bai was incorrectly not listed as the corresponding author. The correct version is shown here.

The Royal Society of Chemistry apologises for these errors and any consequent inconvenience to authors and readers.

## Supplementary Material

